# Patients with sickle-cell disease exhibit greater functional connectivity and centrality in the locus coeruleus compared to anemic controls

**DOI:** 10.1016/j.nicl.2019.101686

**Published:** 2019-01-22

**Authors:** Ravi R. Bhatt, Lonnie K. Zeltzer, Julie Coloigner, John C. Wood, Tom D. Coates, Jennifer S. Labus

**Affiliations:** a UCLA Pediatric Pain and Palliative Care Program, Division of Hematology-Oncology, Department of Pediatrics, David Geffen School of Medicine at UCLA, Los Angeles, CA, USA; b Center for Neurobiology of Stress and Resilience, Department of Medicine, Vatche and Tamar Division of Digestive Diseases, David Geffen School of Medicine at UCLA, Los Angeles, CA, USA; c Childrens Hospital Los Angeles, Department of Radiology, Los Angeles, CA, USA; d Childrens Hospital Los Angeles, Department of Cardiology, Los Angeles, CA, USA; e Childrens Center for Cancer, Blood Disease and Bone Marrow Transplantation, Children's Hospital Los Angeles (CCCBD), Los Angeles, CA, USA

## Abstract

• Patients with sickle-cell disease (SCD) have greater resting-state functional connectivity between the locus coeruleus (LC) and dorsolateral prefrontal cortex (dlPFC).
• Patients with SCD have greater resting state centrality of the LC
• SCD patients with chronic pain exhibited even greater functional connectivity between the LC and dlPFC.
• This study supports hyper-connectivity between the LC and PFC is a potential chronic pain generator.

## 1 Introduction

Sickle Cell Disease (SCD) affects between 90,000–100,000 Americans (Smith et al., 2008) and is associated with significant morbidity and mortality. SCD is caused by a mutation in the human beta-globin gene, resulting in polymerization of the oxygen transporting protein haemoglobin (hemoglobin(Hb)S) in the deoxy state. The polymerization of HbS under low oxygenation conditions causes red blood cells (RBCs) to become rigid and sickle-shaped, increasing the risk of vascular occlusion, ischemia and vaso-occlusive pain crises (Piel et al., 2017; Rees et al., 2010). Under adverse conditions, damaged endothelium from sickle-shaped RBCs contributes to vaso-occlusion, (Platt et al., 1984) infarctions, haemolysis, vasculopathy and cerebrovascular disease (Rees et al., 2010).

Brain imaging studies (Balci et al., 2012; Chen et al., 2017; Schatz and Buzan, 2006; Scott Mackin et al., 2014) have shown atrophy of the corpus collosum, frontal lobes, thalamus, and basal ganglia, in addition to increased resting-state functional connectivity (RS-FC) of the default-mode network (DMN) in children, that was associated with cognitive decline (Chen et al., 2017; Colombatti et al., 2016; Schatz and Buzan, 2006; Scott Mackin et al., 2014). Increased amplitude of low-frequency fluctuations (ALFF) in the orbitofrontal cortex, anterior cingulate cortex (ACC) and posterior cingulate (PCC) compared to anemic controls has also been observed (Coloigner et al., 2017). These cortical abnormalities may play key roles in rewiring central pain pathways and causing neuroplastic alterations, resulting in vulnerability for development of neuropathic pain (Brandow et al., 2013; Darbari et al., 2015; Dias Antunes et al., 2017; Gustin et al., 2012). Neuropathic pain resulting from dysfunction of the central nervous system (CNS) has been shown to underlie SCD (Ballas et al., 2012; Brandow et al., 2014; Darbari et al., 2015; Dias Antunes et al., 2017; Wang et al., 2010) via screening tools(Brandow et al., 2014; Wilkie et al., 2010) and laboratory pain testing,(Brandow et al., 2013; Jacob et al., 2015; O'Leary et al., 2014) with as many as 40% of patients with SCD reporting neuropathic pain.(Brandow et al., 2014).

Animal models of neuropathic pain have demonstrated that a region in the brainstem called the locus coeruleus (LC) and its central ascending noradrenergic (NA) projections can facilitate and exacerbate non-SCD neuropathic pain (Aston-Jones and Cohen, 2005; Kaushal et al., 2016; Szabadi, 2013). LC axons project to the thalamus, anterior cingulate (ACC), hippocampus, hypothalamus, amygdala, and medial prefrontal cortex (mPFC). In particular, the LC innervates the mPFC(Aston-Jones and Cohen, 2005) with respect to changes in this corresponding (LC-dlPFC) circuit. This might contribute to the neuropathic pain observed in patients with SCD, a region activated by nociceptive input when transitioning from acute to chronic pain and central sensitization in humans (Baliki et al., 2012; Kaushal et al., 2016). The LC-mPFC circuit dysregulation is triggered in rodents by constant noxious input (Kaushal et al., 2016). This shifts noradrenaline activity to create a predominance of facilitative α1-adrenoreceptors rather than inhibitory α2-adrenoreceptors, (Kaushal et al., 2016) contributing to hypersensitivity (Martins et al., 2015, 2013). From a RS-FC perspective, the mPFC in rodents strongly parallels the dorsolateral prefrontal cortex (dlPFC) in primates with respect to executive function, attention and decision making (Dalley et al., 2004; Leonard, 2016). Overall, findings support that processes involving the mPFC in rodents are served by the vmPFC and dlPFC in primates (Eichenbaum, 2017). As dysregulation of the LC-mPFC circuit can facilitate pain hypersensitivity in rodents, (Kaushal et al., 2016; Taylor and Westlund, 2017) changes in this corresponding circuit might contribute to the neuropathic pain observed in patients with SCD (Brandow et al., 2014; Dias Antunes et al., 2017).

The aim of this study was to investigate RS-FC of the LC in patients with SCD compared to patients having anemia without chronic pain (anemic controls, or AC) using neuroimaging, seed-based and graph theory connectivity analyses. The seed-based resting-state connectivity analysis allowed us determine where connectivity differences were present in precise regions, while the graph-theory based connectivity analysis allowed us to determine what differences were present in network influence (centrality) in respect to the LC. We hypothesized that patients with SCD would show greater functional LC connectivity and centrality with other key brain regions that play roles in chronic pain compared to ACs.

## 2 Methods

### 2.1 Participants

African-American (*N* = 14), Hispanic (*N* = 3), and Middle Eastern (N = 1) patients with SCD along with Asian (*N* = 6), and White (N = 6) anemic controls were recruited from the Children's Hospital Los Angeles (CHLA) hemogloinopathy center. All procedures were approved by the institutional review board (IRB) and all participants provided written informed consent. A total of 12 anemic controls (mean age = 24.2, SD = 7.4, 8 females) and 18 patients with SCD (mean age = 21.2, SD = 8.6, 7 females) were included after quality control of the MRI data. Eight patients with SCD and ten ACs were undergoing transfusions. In the SCD population, 14 patients had hemoglobin SS and 4 hemoglobin SC disease. Three of the SCD patients exhibited chronic widespread pain. In the AC population, 2 patients had *E*-beta thalassemia, 4 patients had Beta Thalessemia Major, 2 patients had Hereditary Spherocytosis, 1 patient had Congenital Dyserythropoietic Anemia, 1 patient had Autoimmune Hemolytic Anemia, 1 patient had Thalessemia Intermedia, and 1 patient had Hemoglobin H Constant Spring. No patients with AC exhibited chronic pain. A subset of 8 sickle cell patients had been recieving regular 2-week transfusions for at least a year to reduce hemoglobin SS concentration and prevent progression of vascular disease. Regular transfusions were scheduled to keep the percent of hemoglobin SS <30% in hopes of reducing strokes. This stops intermittent vaso-occlusive pain crises, but does not affect chronic regional pain syndromes. Sub-analyses of this cohort compared to SCD patients without transfusions did not change the outcome findings. Young adults with previous overt strokes or known cerebrovascular disease were not included in the study. Other exclusion criteria included pregnancy, occurrence of acute chest pain or pain crisis hospitalization in the past month, and additional diagnosed conditions such as epilepsy or traumatic brain injury. SCD patients who had chronic pain (*N* = 3) were identified by the patient's hematologist and co-author (TC) based on clinical status and characteristics of their pain consistent with ongoing neuropathic pain (burning, allodynia, swelling of limb, non-response to opioids, etc.) lasting >6 months. These data were used to perform an additional exploratory analysis. All patients were in their steady-state, and no focal neurologic deficits were documented in their medical records.

### 2.2 Clinical variables

In addition to recording age, gender, and body mass index (BMI), a complete blood count (CBC) panel was recorded on all patients.

### 2.3 Imaging acquisition

All imaging was conducted on a 3 T Phillips Achieva. After careful positioning of the subject, padding of the head to reduce movement, and application of noise-reducing headphones, a standard high-resolution T1-weighted 3D structural scan, covering the whole brain (160 sagittal slices) was obtained; (TE: 8.20 ms, TR: 3.77 ms, flip angle: 8 degrees, in-plane resolution: 256 mm × 256 mm, FOV: 256 × 224 mm, percent phase FOV: 87.5, slice thickness/gap: 1/1 mm). Afterwards, an 8-min resting state functional scan (26 axial slices) was obtained (TE: 50 ms, TR: 2000 ms, flip angle: 90 degrees, in-plane resolution 2.3 mm × 2.3 mm, FOV: 220 mm × 220 mm, slice thickness/gap: 5/0 mm). Subjects were instructed to close their eyes, not think of anything, not fall asleep, and keep as still as possible.

### 2.4 Image pre-processing and quality control

Imaging data were processed using SPM12. Preprocessing for quality control included bias-field correction, co-registration, motion correction, spatial normalization, tissue segmentation, and Fourier transformation. Structural images were included in subsequent analyses based on compliance with acquisition protocol, full brain coverage, minimal motion, Gibbs ringing, absence of flow/zipper and minor atrophy/vascular degeneration. Functional imaging pre-processing included transformation from DICOM into NIFTI, slice-time correction, co-registration with high-resolution structural images, spatial normalization into MNI space, realignment to correct for subject movement and unwarping to correct for the movement-by-distortion interaction (using Unwarp in SPM12), and resampled to a voxel size of 2 × 2 × 2 mm. Functional images were included in subsequent analyses based on compliance with acquisition protocol, full brain coverage, motion estimate of <2 mm in the three directions of translation and three directions of rotation, minimal signal loss, and proper co-registration and normalization with the structural image.

### 2.5 Functional network construction

The normalized resting-state functional images were then processed using the CONN 17 toolbox in MATLAB (Whitfield-Gabrieli and Nieto-Castanon, 2012). Resting-state images were filtered using a band-pass filter (0.001 Hz/<f < 0.01 Hz) to reduce low and high frequency noise. A component-based noise correction method, CompCor (Whitfield-Gabrieli and Nieto-Castanon, 2012), was used to remove motion artifacts - including six motion realignment parameters, as well as confounds for white matter and cerebrospinal fluid (CSF) - for better sensitivity and specificity of the analysis. Gray matter images for each subject were segmented using the Destrieux (cortical) and Harvard-Oxford Subcortical Atlases (Destrieux et al., 2010; Irimia et al., 2012) and parceled into 165 cortical and subcortical regions. Two additional regions of interest (ROIs) consisting of 2 mm spheres located at the bilateral LC were manually created in MarsBar based on the recommended location of the LC (MNI coordinates: X = ±4, Y = −36, Z = −24) (Brett et al., 2002; Keren et al., 2009). This process resulted in a 167 × 167 adjacency matrix for each subject. ROI-to-ROI functional connectivity – defined as cross correlations of all ROI's blood-oxygen-level dependent (BOLD) time series – were computed in the CONN toolbox and then Fischer transformed to create *Z* values. The connectivity between the 167 brain regions was indexed by a matrix of Fisher *Z* transformed correlation coefficients reflecting the association between average temporal BOLD time series signals across all voxels in each brain region. Functional connections were retained at *Z* ≥ 0.3 and all values below that threshold were set to 0. The magnitude of the z-score represents the weights in the functional network. As a sensitivity analysis, absolute thresholding was also applied at 0.2, 0.4, 0.5, 0.6, 0.7, and 0.8 (Van Den Heuvel et al., 2017).

### 2.6 Computing network metrics

Thresholded single-subject functional networks were then used to calculate network metrics using in-house scripts and Graph Theory Toolbox (GTG) in MATLAB (Spielberg, 2014). Measures of centrality including degree, strength, betweenness centrality and eigenvector centrality were computed. Regions with high centrality are highly influential and communicate with many other regions, facilitate functional integration, and play a key role in network resilience to insult (Rubinov and Sporns, 2010). Indices of centrality included (1) *Degree:* reflecting the number of connections to an ROI, (2) *Strength:* reflecting the weighted version of the number of connections to an ROI*,* (3) *Betweenness Centrality:* reflecting the ability of an ROI to control information flow and modulate information (act as a bridge) between two other modules, and (4) *Eigenvector Centrality:* reflecting the global prominence of the region due to connections with other highly connected nodes Rubinov and Sporns, 2010).

### 2.7 Network metrics statistical analyses

To determine group differences in network metrics, GLM-based non-parametric permutation testing was performed, controlling for age and sex, at 5000 iterations in MATLAB. Permuted probability values were further corrected using the false discovery rate (FDR) to calculate significance at *p*_(FDR)_ < 0.05.

### 2.8 Seed-to-voxel functional connectivity analysis

**Fig. 1:**
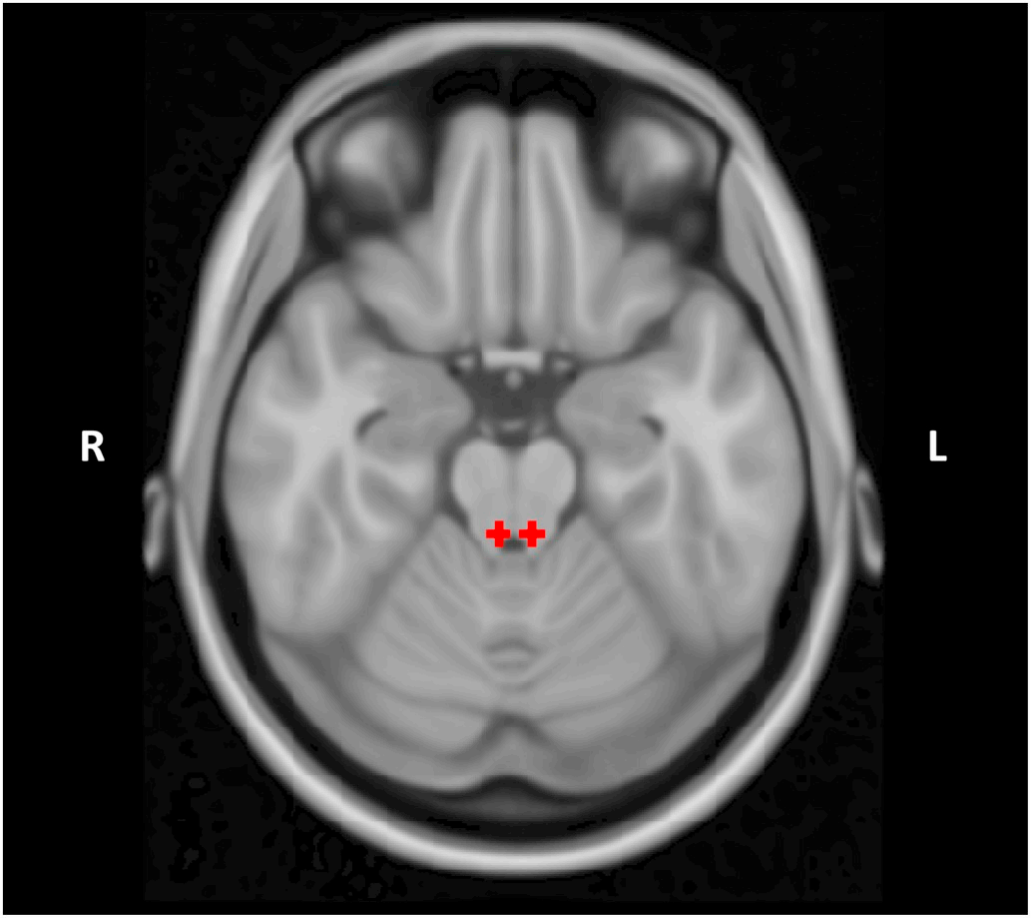
Bilateral spherical seeds of 2 mm each placed in the locus coeruleus.

The 2 mm spherical seeds in the bilateral LC (Fig. 1) were used as seeds in CONN 17 for a seed-to-voxel whole-brain connectivity analysis. Age and sex were included as covariates. The resulting single-subject beta maps were extracted and smoothed at 4 mm full-width half maximum (FWHM). To compare differences between groups, a non-parametric independent sample *t*-test was conducted using Statistical nonParametric Mapping (Nichols and Holmes, 2001), specifying 5000 permutations with variance smoothing applied at 4 mm FWHM. The initial cluster-forming threshold was set at *p* < .001 and significance level was set at *p*_(FWE)_ < 0.05. To determine the effect of chronic transfusion status on the results, we repeated the analyses including an indicator variable representing whether a subject was receiving blood transfusions as a covariate. Additionally, as an exploratory analysis within patients with SCD, we compared 3 patients who actively exhibited symptoms of chronic pain to patients who did not actively exhibit chronic pain. This was done by exporting the eigenvalues for the connectivity dyad within Statistical Parametric Mapping (SPM) for every subject and then conducting a Welch's *t*-test between the groups.

### 2.9 Clinical variables

To investigate differences in CBC panel results between SCD and AC, independent sample *t*-tests were conducted along with Cohen's d calculations to measure effect size. Cohen's d, independent of sample size, is used to indicate the magnitude of the difference between two means, and is calculated by subtracting one group mean from another and dividing it by the pooled standard deviation. A value of d < 0.5 is considered a small effect, 0.5 < d < 0.8 a medium effect, and d > 0.8 a large effect (Sullivan and Feinn, 2012).

## 3 Results

A summary of the clinical variables – including age, gender, vital signs and CBC results – can be seen in Table 1. There was no significant difference in age, sex, body habitus, or blood pressure between groups. As expected, patients with SCD had higher white blood cell counts, lower RBC counts, lower hematocrit percentages and higher reticulocyte counts compared to anemic controls. See Table 1.

**Table 1:** Summary of demographic and clinical variables.

	SCD (*N* = 18, 8 F)	ACTL (*N* = 10, 7 F)	*t*	*p*	*d*
	Mean (SD)	Mean (SD)			
Age (yrs)	21.2 +/− 8.3	25.5 +/− 7.5	−1.41	0.17	−0.54
Height (cm)	161.6 +/− 11.3	162.8 +/− 10.4	−0.04	0.97	−0.11
Weight (kg)	57.3 +/− 9.5	60.5 +/− 17.3	−0.62	0.54	−0.23
BMI	23.0 +/− 5.9	22.1 +/− 2.3	0.55	0.59	0.20
SBP	111.3 +/− 14.2	111.5 +/− 10.3	−0.40	0.97	−0.24
DBP	62.5 +/− 8.1	62.8 +/− 9.0	−0.13	0.89	−0.03
WBC (K/mcL)	10.3 +/− 4.6	6.6 +/− 2.2	2.41	**0.02**	**1.03**
RBC (M/mcL)	3.2 +/− 0.7	3.7 +/− 0.4	−2.16	**0.04**	**−0.87**
Hb (g/dL)	9.8 +/− 1.8	10.0 +/− 1.2	−0.34	0.76	−0.13
Hbs (g/dL)	47.4 +/− 29.3	0.0 +/− 0.0	6.86	**< 0.001**	**1.99**
HbF (g/dL)	6.1 +/− 8.3	1.7 +/− 2.5	2.41	**0.04**	**0.67**
Hct (%)	27.9 +/− 4.4	30.0 +/− 3.0	−1.37	0.18	−0.55
MCV (fL)	89.4 +/− 13.9	81.5 +/− 6.0	1.71	0.10	0.73
Plt (K/mcL)	297.4 +/− 112.7	263.0 +/− 114.1	0.77	0.45	0.30
PltVol (fL)	10.0 +/− 0.8	10.6 +/− 1.1	−1.58	0.13	−0.62
Retic (%)	9.6 +/− 5.9	2.6 +/− 3.2	4.08	**< 0.0001**	**1.47**

Groups: Sickle-Cell Disease (SCD), Anemic Controls (AC), Females (F).

Variables: Body Mass Index (BMI), White Blood Cell count (WBC), Red Blood Cell count (RBC), Heboglobin count (Hb), Hematocrit (Hct), Mean Corpuscular Volume (MCV), Platlet count (Plt), Mean Platlet Volume (PltVol), Reticulocyte Count (Retic).

Statistics: standard deviation (SD), t-value (*t*), p-value (*p*), Cohen's d (*d*).

The bolded numbers represent variables that were significantly different between groups.

### 3.1 Patients with SCD exhibit greater connectivity from LC to dlPFC

**Fig. 2:**
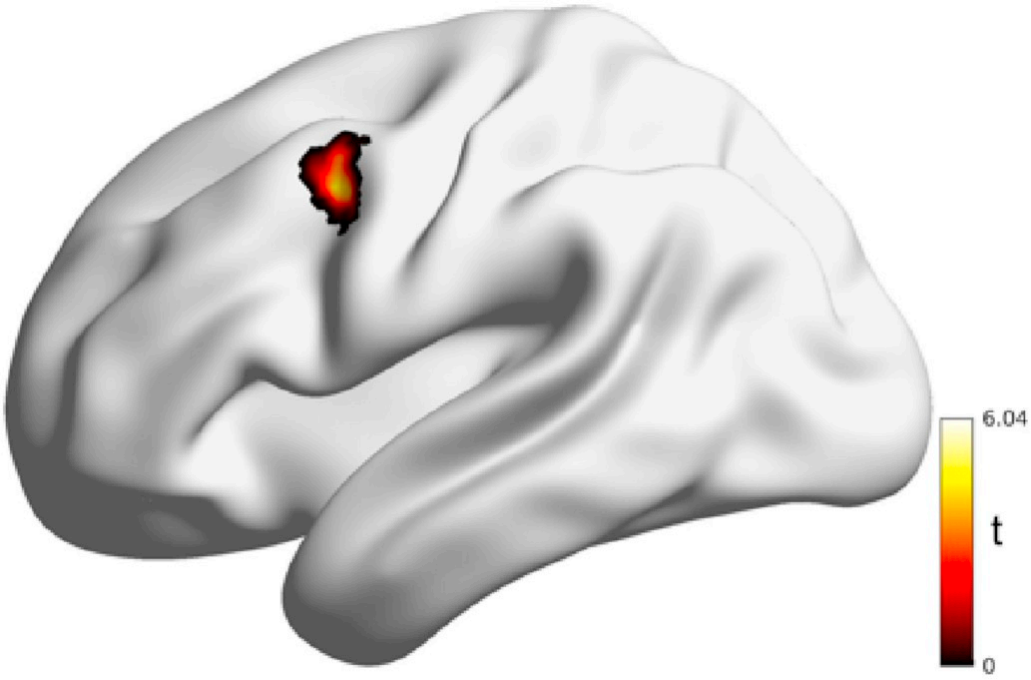
Patients with sickle cell disease exhibit greater connectivity from the left locus coeruleus to the left dorsolateral prefrontal cortex compared to anemic controls.

**Fig. 3:**
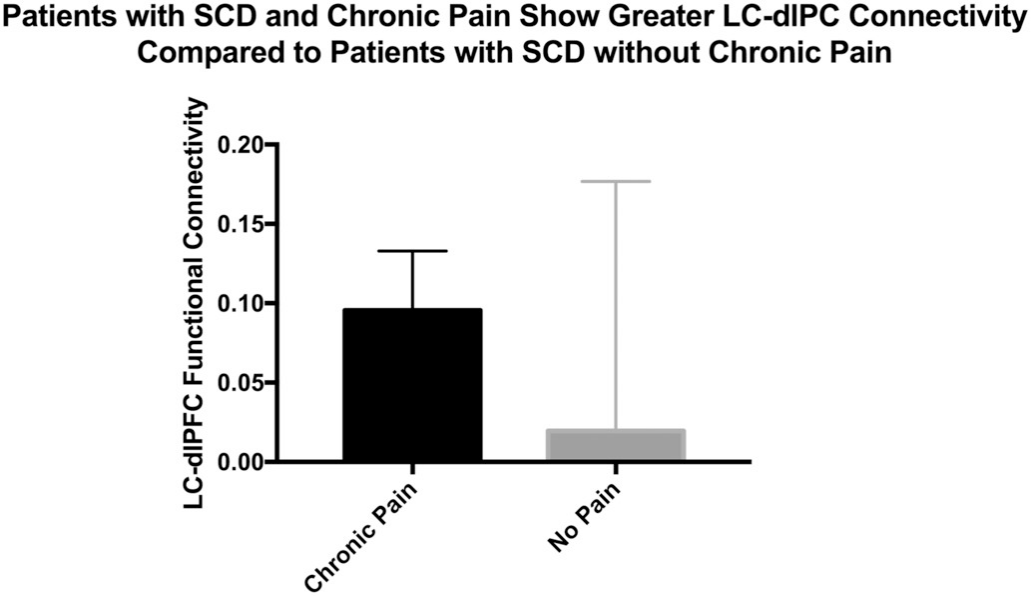
Sickle-cell disease patients with chronic pain exhibit trending greater connectivity between the Locus Coeruleus and Dorsolateral Prefrontal Cortex compared with sickle-cell disease patients without chronic pain (*t*_(14.18)_ = 1.99, *p* = .06, *d* = 0.66).

Results from the seed-to-voxel analysis revealed that patients with SCD had greater connectivity between the left LC and left dorsolateral prefrontal cortex (dlPFC). Specifically, connectivity results showed patients with SCD had increased connectivity between the left LC and left dlPFC (Sallet et al., 2013). No other difference in LC connectivity was observed. When using transfusions as a covariate, the results still showed significant increased connectivity from the left LC to left dlPFC in the SCD patients. Additionally, patients with SCD with chronic pain had a *trend* towards greater LC-dlPFC connectivity compared to patients with SCD without chronic pain (*t*_(14.18)_ = 1.99, *p* = .06, *d* = 0.66), although this result did not achieve statistical significance). See Table 2, Table 3, Fig. 2, Fig. 3.

**Table 2:** Seed-to-Voxel Analysis from Left Locus Coeruleus to Whole-Brain. XYZ represent MNI-Coordinates.

Contrast: SCD > AC						
Seed: left locus coeruleus						
Region	Voxels	X	Y	Z	*t*	*p*_(FDR)_
Left Middle Frontal Gyrus (Left dlPFC)	57	−42	6	44	5.59	0.03

Statistics: t-value (*t*), *p*-value corrected for family-wise error *p*_(FWE)_ < 0.05.

**Table 3:** Seed-to-Voxel Analysis from Left Locus Coeruleus to Whole-Brain with Transfusions as a covariate. XYZ represent MNI-Coordinates.

Contrast: SCD > AC - covariate: transfusions						
Seed: left locus coeruleus						
Region	Voxels	X	Y	Z	*t*	*p*_(FDR)_
Left Middle Frontal Gyrus (Left dlPFC)	26	−50	14	36	4.41	0.04

Statistics: t-value (*t*), *p*-value corrected for family-wise error *p*_(FWE)_ < 0.05.

### 3.2 Patients with SCD exhibit greater centrality of the locus coeruleus

**Fig. 4:**
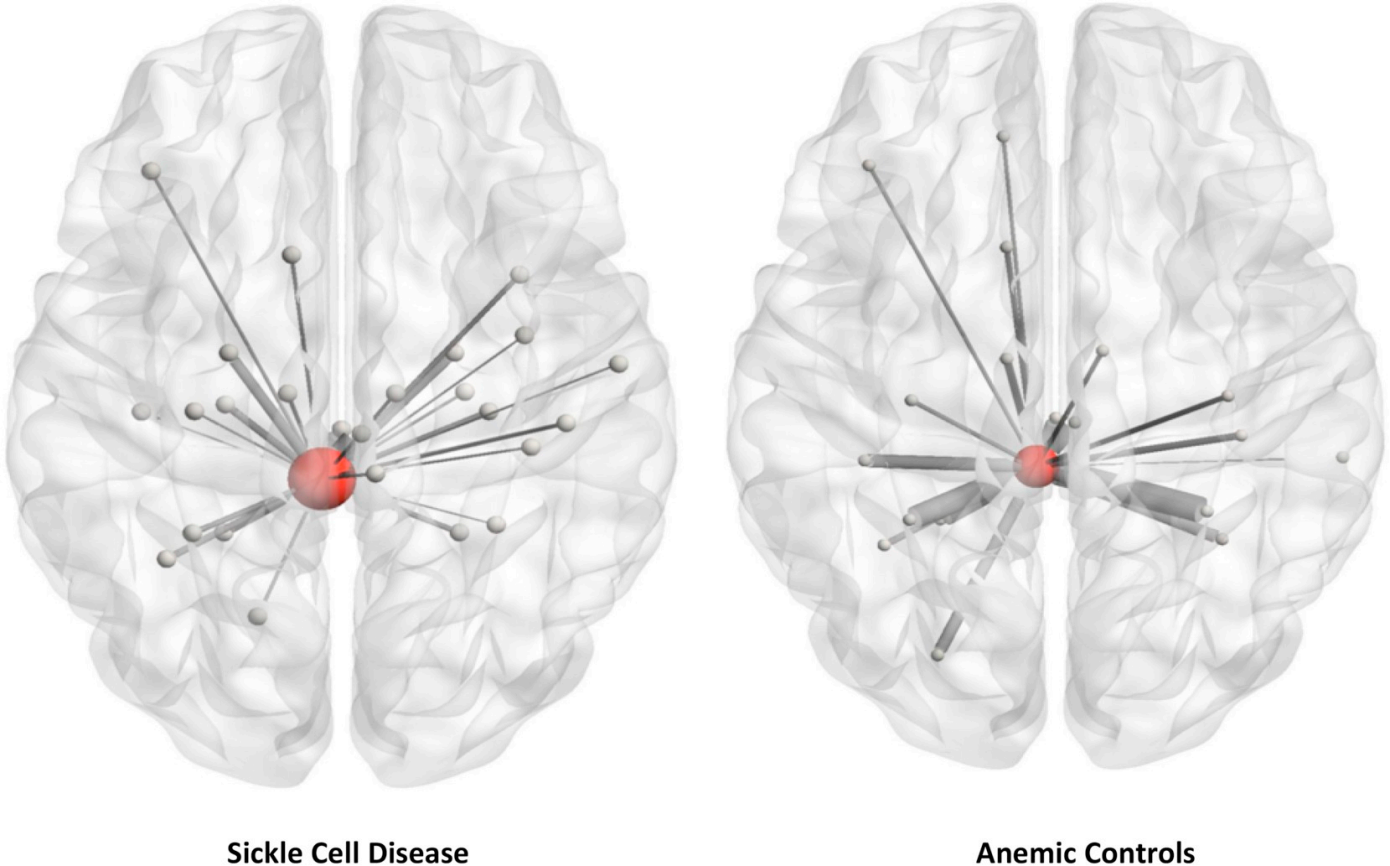
Patients with SCD exhibit greater betweenness centrality in the left Locus Coeruleus compared to Anemic Controls.

Results from the network metrics analysis revealed that patients with SCD exhibited greater *betweenness centrality* in the left LC and greater *strength* in the right LC. These results were consistent across some (Z = 0.2, 0.3, 0.5) but not all threshold levels, showing significant results when observing denser networks. See Table 4 and Fig. 4.

**Table 4:** Summary of network metric results comparing patients with sickle-cell disease and anemic controls, controlling for the effects of age and sex.

Betweenness centrality						
ROI	Threshold	Variable	*t*_*(29)*_	*p*(FDR)	*B*	Interpretation
L_LC	0.2	Group	3.17	0.005	10.06	SCD ↑ AC ↓
L_LC	0.3	Group	2.95	0.006	6.421	SCD ↑ AC ↓

Strength						
ROI	Threshold	Variable	*t*_*(28)*_	*p*(FDR)	*B*	Interpretation
R_LC	0.5	Group	2.176	0.039	0.655	SCD ↑ AC ↓

Groups: Sickle Cell Disease (SCD), AC (Anemic Controls).

ROIs: Left Locus Coeruleus (L_LC), Right Locus Coeruleus (R_LC).

Abbreviations: Betweenness Centrality (BWC).

Statistics: Network construction variable (Pearson's r vs Fisher transformed Z), Beta (*B),* t-value with 29 degrees of freedom (*t(*_*29)*_), Cohen's d (*d)*, *p*-value corrected for false discovery rate *p*_(FDR)_ < 0.05

## 4 Discussion

As hypothesized, group differences in functional connectivity of the LC were found in patients with SCD compared to anemic controls using seed-to-voxel and graph theoretical methods examining LC-based connectomes. Past brain imaging research has found that patients with S.CD with high pain intensity have greater RS-FC in pronociocptive areas such as the ACC, somatosensory cortex, and insula, (Darbari et al., 2015) as well as greater connectivity from the insula, basal ganglia and periaqueductal gray (PAG) brainstem regions to various resting state networks (Case et al., 2017). These patients have also been shown to have increased EEG activity in pain processing regions (Case et al., 2018). Case et al.'s(Case et al., 2017) finding of greater connectivity in the PAG runs parallel to our findings, as there is considerable evidence that the LC receives input from PAG neurons (Cedarbaum and Aghajanian, 1978; Lee et al., 2005; Luppi et al., 1995; Simson, 2001), and plays a large role in the stress response (Borelli et al., 2005; Kiyokawa et al., 2005). Future studies looking at the influence of brainstem activity - including the PAG and LC - and neurochemistry would serve to be beneficial in understanding how activity in the mid-brain influences cortical processing and its influence on pain.

### 4.1 Differences in connectivity of the locus coeruleus in patients with SCD

Patients with SCD compared to ACs had greater LC betweenness centrality – indicating that this region is critical for mediating communication between other brain regions (Rubinov and Sporns, 2010; Sporns, 2013) – and greater LC-dlPFC connectivity. These alterations in LC circuitry run parallel to recent findings in rodent models of neuropathic pain (Kaushal et al., 2016; Marzo et al., 2014). The LC is the primary brain region involved in the synthesis of noradrenaline, (Dahlström and Fuxe, 1964; Jones and Yang, 1985; Westlund et al., 1983) and is the sole source of cortical noradrenaline to the neocortex (Berridge and Abercrombie, 1999; Berridge and Waterhouse, 2003). After exposure to a stressor (e.g. pain), noradrenaline is released as a messenger to various CNS cells with adrenoreceptors. Many projections from the noradrenergic locus coeruleus are to regions that are responsible for regulating the autonomic response to stress, arousal, and homeostatic mechanisms (e.g. suppression of the baroreceptor reflex) (Hwang et al., 1998; Samuels and Szabadi, 2008; Shih and Chan, 1995), all of which can be a risk factor for vaso-occlusive pain crises in patients with SCD who have greater pain sensitivity (Brandow et al., 2013; Coates et al., 2018).

Providing tentative support for the idea that hyperconnectivity of the LC-dlPFC circuit may be present in chronic pain, the three SCD patients suffering from chronic pain had greater functional connectivity between the LC and dlPFC compared to SCD without pain (Cohen's d = 0.66). Although compelling, this hypothesis requires further systematic study in a larger sample using systematic assessments of chronic pain as well with assessment of frequency of vaso-occlusive crises.

Additionally, since SCD patients overall had greater LC-dlPFC connectivity compared to anemic controls, this hyperconnectivity could possibly be a precursor to the development of vaso-occlusive crises or of chronic pain via a hyperactive autonomic nervous system (discussed in the next section). We pose these hypotheses for testing.

Moreover, the dlPFC is a core region in the executive control network that is involved in working memory, cognitive flexibility, selective attention and response inhibition (Elliott, 2003). One might speculate that the observed alterations in dlPFC_LC functional connectivity might underlie executive function deficits and associated cortical changes reported in SCD (Chen et al., 2017; Coloigner et al., 2017; Downes et al., 2019, Downes et al., 2018; Schatz and Buzan, 2006; Scott Mackin et al., 2014; Swift et al., 1989; Vichinsky et al., 2010). High levels of noradrenaline via projections from the LC to the prefrontal cortex have been shown to impair prefrontal inhibitory functions critical for executive function (Xing et al., 2016). Further work is need to examine this possibility.

### 4.2 Implications of the findings for the autonomic nervous system and blood flow

Our findings also suggest clinically relevant outcomes, such as the possibility that peripheral blood-flow may be altered due to hyperconnectivity of the LC and dlPFC. Since arterioles are innervated by sympathetic neurons, increased microvascaular transit time due to increased sympathetic activity – which the LC modulates (Benarroch, 2009) – can result in increased vaso-occlusion (Coates et al., 2018). Acute stressors, such as pain, induce noradrenaline release in the PFC in rodents (Jett and Morilak, 2012; Marzo et al., 2014), and continuous release can result in allostatic stress overload (McEwen, 2004) and subsequent vasoconstriction via increased sympathetic nervous system activity (Connes and Coates, 2013; McEwen and Seeman, 2009), a hallmark feature of SCD (Connes and Coates, 2013). Peripheral noradrenaline release is exacerbated by the infarcted tissue damage and inflammation caused by the sickled RBCs. This peripheral effect causes more tissue ischemia, further release of noradrenaline, and leads to a debilitating top-down and bottom-up pain cycle (Ballas, 2005). We compared the SCD group to the anemia controls with and without those who were on transfusion protocols and the findings did not change. This lack of effect of transfusion would suggest that there are other differences between non-SCD controls and patients with SCD that might influence central pain processing that are independent of level of hemoglobin. These differences await further study. Humans with sickle trait as well as sickle cell anemia have autonomic dysfunction of unknown cause and these abnormalities are independent of transfusion (Khaleel et al., 2017; Sangkatumvong et al., 2011). Direct measures of nerve impulse responses to pain are greater in SCD mice than wild type. Nerve conduction abnormalities have been seen in mice as well (Cataldo et al., 2015a). The findings in the present work uncover yet another pain-related alteration in SCD subjects that is not present in control. The mechanism of this neural hypersensitization remains elusive. Increased sympathetic activity, or parasympathetic withdrawal, (Alexy et al., 2010; Connes and Coates, 2013) may be an underlying mechanism. However, future studies investigating neurotransmitters such as GABA and noradrenaline in the locus coeruleus and dlPFC, using imaging techniques such as diffusion tensor imaging, PET and spectroscopy along with measures of pain sensitivity and vasoconstriction, would be crucial to establish causal mechanisms in humans.

### 4.3 Potential significance and therapeutic implications

There appears to be neurological differences in patients with SCD that may play a role in pain mediation. Neurological hypersensitivity has been observed in SCD subjects in response to respiratory control (Sangkatumvong et al., 2011) and pain in humans, (Khaleel et al., 2017) and in response to pain in transgenetic mice (Cataldo et al., 2015b). Thus, there may be some effect of this disorder on neural systems that plays a significant role in vascular control and pain. We now show differences in brain connectivity in patients with SCD, and importantly, have used anemic subjects as controls so these differences are not due to anemia.

Future studies investigating potential therapeutic pain prevention strategies or targeted analgesic treatments for chronic pain in SCD should monitor the level of the LC centrality in neural connectivity. The goal would be to reduce noradrenaline release and the strength of connectivity of the LC with the dlPFC. Such changes could enhance prefrontal cortical control by shifting prefrontal processes. For example, activity in the ventromedial prefrontal cortex (vmPFC) has been shown to be involved in the process of resilience and positive behavioral adaptation in the face of adversity or trauma (Hänsel and Von Känel, 2008) Increased vmPFC activity has also been shown to be associated with reduced pain (Woo et al., 2015). Future studies investigating more nuanced prefrontal cortical activity in chronic pain are necessary to develop therapeutic approaches that can have a beneficial effect. From a pharmacological perspective, strategies that increase the role of relevant LC-mediated neurotransmitters involved in pain inhibition might be considered. For example, tricyclic anti-depressants and noradrenergic reuptake inhibitors (NRIs) facilitating activity at alpha 2-adrenoreceptors have been shown to lose analgesic efficacy over time, (Llorca-Torralba et al., 2016) a finding that may be related to enhanced facilitative alpha 1-adrenoceptor activity (Taylor and Westlund, 2017) with continued pain. This latter finding suggests that current pharmacological treatments may be able to slow down the development of chronic pain, but treatments aimed at diminishing alpha-1 activity would be crucial to terminating it. Excessive alpha-1 noradrenaline activity in the PFC has been associated with stress-induced cognitive impairments, such as poor attention regulation and disinhibited behaviors (Birnbaum et al., 1999). Thus, it is hypothesized that reducing stress-related increased connectivity within the LC-PFC pathway would improve chronic pain symptoms.

At the level of the dlPFC, various non-invasive treatments such as repetitive transcranial magnetic stimulation (rTMS) and transcranial direct current stimulation (tDCS) have been shown to be effective in patients with chronic pain (Brighina et al., 2004; Conforto, 2014; Umezaki et al., 2016) who also have psychological symptoms, such as depression. However, no published reports currently exist with regards to patients with SCD (Seminowicz and Moayedi, 2017). Mindfulness meditation and hypnosis are other non-pharmacological treatments shown to be effective in improving blood flow, reducing activity in the dlPFC, and decreasing pain sensitivity (Barrett et al., 2016; Bhatt et al., 2017; Jensen et al., 2014; Jensen and Patterson, 2014; Zeidan et al., 2015, 2012). It would be useful to test strategies that alter this neural pathway in patients with SCD. While treatment of acute pain crises in patients with SCD is crucial for quality of life and survival (e.g. acute chest syndrome), the findings of this study suggest that strategies that alter the strength of the LC-dlPFC connectome prior to the chronification of pain would have significant impact for individuals living with SCD. Such targeted research would also strengthen our understanding of mechanisms involved in the transition from acute to chronic pain.

## 5 Conclusions

The findings of the current study support greater connectivity of the LC-dlPFC circuit, along with a connectome showing a greater amount of information flow in the LC to other parts of the brain in patients with SCD. These are the first findings we know of that establish this LC-dlPFC hyperconnectvity link in humans with SCD and provide the basis for further investigation of cellular mechanisms such as noradrenaline activity underlying this link, the modulatory role that the LC may play in altering other brain networks, and associating it with chronic pain symptoms and physiological activity in the periphery. Additionally, it provides the basis for investigating methods of treatments to regulate dysfunctional noradrenaline activity originating at the LC along with non-pharmacological treatments such as TMS, tDCS, meditation, and hypnotherapy aimed at diminishing activity in the dlPFC and reducing painful symptoms.
